# Nucleoside drives the self-assembly and enables delivery of phosphate containing therapeutics

**DOI:** 10.1016/j.mtbio.2026.103556

**Published:** 2026-08-13

**Authors:** Jiamin Yao, Liu Liu, Junfeng Wang, Kaichao Wang, Jiang Liu, Yao Yuan, Hang Zhao

**Affiliations:** State Key Laboratory of Oral Diseases & National Center for Stomatology & National Clinical Research Center for Oral Diseases & Research Unit of Oral Carcinogenesis and Management & Chinese Academy of Medical Sciences, West China Hospital of Stomatology, Sichuan University, Chengdu, Sichuan, 610041, China

**Keywords:** Self-assembly, Supramolecular, Nucleoside, Nanoparticle, Drug delivery

## Abstract

Nucleobase driven molecular recognition has enabled the self-assembly of a wide range of nucleosides and nucleotides. Emerging evidence suggests that ribose actively influences the nucleoside self-assembly process; however, how ribose associated chemical features contribute to this process remains unclear. Here, we propose a previously underexplored nucleoside phosphate recognition model in which the ribose 5′-OH group likely participates in hydrogen bonding with phosphate moieties, assisted by electrostatic pre-organization through protonation deprotonation equilibria. This model describes a site specific and reversible recognition mode that operates outside canonical base pairing within the systems examined. As a proof of concept, we systematically examined a series of phosphate-containing molecules, including nucleoside monophosphates, non-nucleosidic phosphate esters, and nucleoside analog drugs. Isoguanosine (isoG), adenosine (A), and guanosine (G) formed stable supramolecular assemblies with these phosphate containing molecules in aqueous solution, supporting the extendability of this assembly strategy across the tested systems. In particular, isoG organized nucleoside analog drugs and oligonucleotide RNAs into stable yet dynamic supramolecular complexes without chemical modification. These assemblies improved the biological activity and stability of phosphate containing nucleoside analogues, and provided preliminary evidence for oligonucleotide based applications. Together, these findings support a plausible nucleoside phosphate recognition model and provide a modular supramolecular strategy for constructing carrier free nucleoside based nanomaterials.

## Graphical abstract

**Figure:**
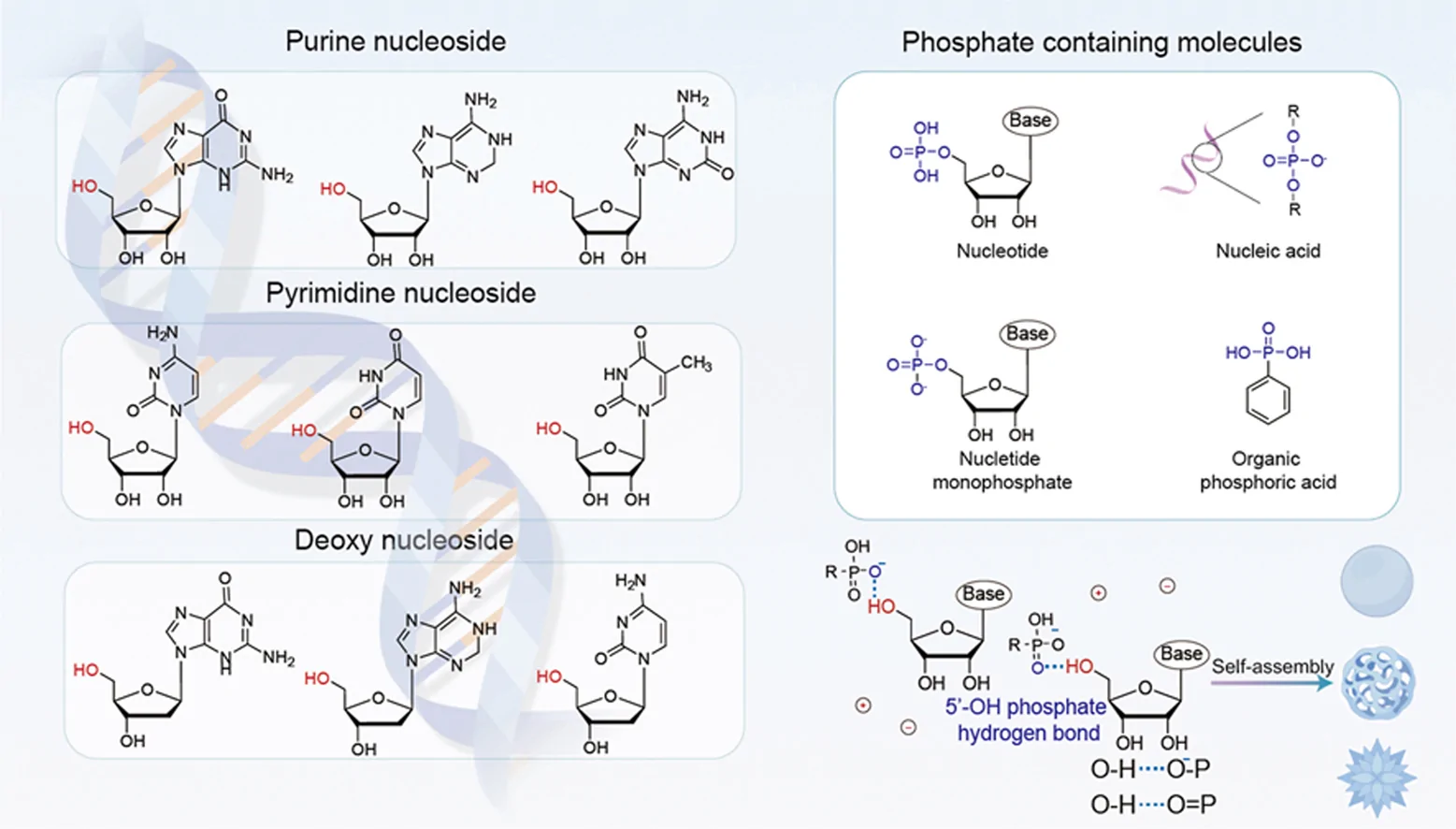

## 1 Introduction

Nucleotide and nucleoside self-assembly is a spontaneous process driven by multiple non-covalent interactions. Key mechanistic principles including Watson-Crick base pair, Hoogsteen hydrogen bonds, π-π stacking, hydrophobic interactions and metal coordination, which organize structurally defined biomolecular units into functional materials [[1], [2], [3]]. Leveraging these mechanisms, researchers have constructed controllable self-assembly systems such as DNA origami, G-quadruplexes, and nucleoside hydrogels [4,5]. Owing to their biocompatibility, programmability, and stimulus responsiveness, these assemblies have demonstrated significant potential in biosensing, drug delivery, and tissue engineering [5,6]. For decades, nucleobase coded molecular recognition has been regarded as the central determinant of nucleoside and nucleotide self-assembly, while considering the ribose sugar as a passive scaffold.

However, this base centric paradigm is increasingly challenged. Emerging evidence shows that ribose chemical properties, conformational dynamics, and modifications actively influence assembly pathways, structural stability, and functional outcomes. The ribose ring's conformational equilibrium (e.g., C2′-endo vs. C3′-endo) modulates global duplex architecture and base stacking efficiency, while chemical modifications (2′-O-methyl, 2′-fluoro, 4′-CF_3_) fine-tune puckering and affect duplex stability, nuclease resistance, and protein interactions [[7], [8], [9]]. Ribose functional groups, such as 2′,3′-cis diols, can participate directly in dynamic covalent interactions, facilitating higher-order assembly [[10], [11], [12]]. Replacing ribose with alternative sugars or non-natural backbones produces artificial nucleic acids (e.g., TNA) with markedly different assembly behaviors, underscoring ribose's active role in molecular organization [13].

Building on this understanding, our team has long been interested in this inconsistency. Our previous work has demonstrated that ribose hydroxyl groups play a decisive role in supramolecular organization. As early as 2014, we reported that modifications of hydroxyls in guanosine analogues dramatically altered their assembly morphologies, even when nucleobase identity and canonical stacking were preserved. Structural analysis revealed that hydroxyl mediated hydrogen bonding stabilizes specific sugar conformations, reshaping higher order organization [14]. More recently, in 2024, we established that sugar-ring conformational stability, regulated by hydroxyl involved hydrogen bonding and hydration, constitutes an intrinsic determinant of G-quadruplex hydrogel robustness [15]. In these systems, nucleobases are essential for initiating primary assembly, but the persistence, stability, and structural organization of the assembly are predominantly dependent on the ribose framework rather than on base pairing itself.

Here, we propose a previously underexplored 5′-phosphate hydrogen bonding interaction as a molecular recognition motif that enables the co-assembly of nucleosides and phosphate-containing molecules into nucleoside supramolecular nanostructures (NSN). According to our model, this process is electrostatically pre-organized through protonation deprotonation equilibria on nucleobase. This motif mainly relies on hydrogen bonding between the ribose 5′-OH and phosphate groups, providing directional and programmable assembly in aqueous solution. As a proof-of-concept, we constructed NSN using isoG and fludarabine phosphate (F-araAMP), a clinically approved nucleoside analog for hematologic malignancies. The resulting F-araAMP-isoG NSN exhibited well-defined morphologies and improved pharmacokinetics, including prolonged circulation time, reduced clearance, and enhanced tumor accumulation in an oral cancer model. These features led to improved therapeutic efficacy and reduced systemic toxicity compared to free drug administration. More importantly, this emechanism can be further extended to the RNA delivery, as the isoG and RNA can also self-assembly into NSN and promote in vivo delivery effciciency.

Taken together, these findings demonstrate a new nucleotide-based self-assembly system, which offers a potential strategy for deliverying both small and large molecule phosphate containing therapeutics. Furthermore, 5′-phosphate hydrogen bonding is proposed as a previously overlooked molecular recognition motif, providing a supramolecular design principle for nucleoside-based systems. By expanding the repertoire of interactions available for nucleoside-based materials, this work opens new opportunities for dynamic self-assembly and the development of next-generation nucleoside therapeutics.

## 2 Methods and materials

All chemical reagents were available purchased from commercial sources and of pure analytical grade and used without further purification. isoG was purchased from Jiangsu Aikon Biopharmaceutical R&D Co., Ltd. (molecular weight 283.24, purity 98%). F-araAMP was bought from bidepharm (molecular weight 365.21, purity 98%). guanosine(G) was purchased from Sigma-Aldrich (molecular weight 283.24, purity 97%). Adenosine (A) was purchased from TCI chemicals (molecular weight 267.24, purity 99%).

PBS (0.01 M) was purchased from HycloneTM (USA). Cy5 NHS were purchased from MCE (China). 0.1 mL insulin syringe was obtained from Becton, Dickinson and Company China (China). Dulbecco's modified eagle medium (DMEM), fetal bovine serum (FBS), and penicillin–streptomycin liquid were purchased from Gibco (USA). Trypsin and dimethyl sulfoxide (DMSO) were purchased from Sigma-Aldrich (USA).

### 2.1 Measurements F-araAMP-isoG

^1^H and ^31^P Nuclear magnetic resonance (NMR): 10 mg of isoG, F-araAMP, and lyophilized F-araAMP-isoG mixture and F-araAMP-isoG NSN were dissolved by heating with DMSO-*d*_6_ (the isoG/F-araAMP assembly was first prepared in pure water, the resulting aqueous assembly was completely freeze-dried and re-dissolved in DMSO-*d*_6_ for ^1^H NMR analysis), 700 μl of the solution were placed in NMR tubes and cooled at room temperature ^1^H NMR spectra were acquired at 600 MHz using an AV II spectrometer (Bruker, Germany). F-araAMP and F-araAMP-isoG NSN were dissolved by heating with DMSO-*d*_6_, ^31^P NMR spectra were acquired at 600 MHz using the same spectrometer.

Ultraviolet-visible spectroscopy (UV-vis): The UV-vis spectra of all samples were determined on a Perkin-Elmer Lambda 20/2.0 UV-vis spectrophotometer.

Transmission electron microscopy (TEM): The morphology and size of nanoparticle were measured by the TEM (Thermo Fisher, USA) at a voltage of 200 kV. The sample was prepared by dropping the solution (1 mg/mL) onto a carbon-coated copper grid. Then the grid was immersed into liquid nitrogen and freeze-dried in vacuum at −50 °C prior measurement.

Dynamic light scattering (DLS): DLS measurements (Malvern Panalytical, Netherlands) were performed with a 4.0 mW laser operating at λ = 633 nm. All samples were determined at a scattering angle of 90°.

Circular dichroism (CD): The CD spectra were recorded on a JASCO J-810 spectropolarimeter at 25 °C within the range of 100-350 nm with a bandwidth of 2.0 nm, data pitch of 0.1 nm, and scan speed at 20 nm/min.

Powder X-ray diffraction (PXRD): PXRD study was carried out with xerogel of F-araAMP-isoG NSN using an X-ray powder diffractometer (PaNalytical-Empyrean, Netherlands) from an angle range of 10° to 40° at 25 °C.

Fourier Transform Infrared Spectroscopy (FTIR): FTIR spectra (Thermo Fisher, USA) of isoG, F-araAMP, lyophilized F-araAMP-isoG mixture, and F-araAMP-isoG mixture NSN were recorded in the 4000–500 cm^−1^ range using a thermos fisher spectrophotometer.

### 2.2 Construction of NSN

For isoG, pyrimidine nucleotide, deoxynucleoside with phosphate-containing molecules:the reaction dose was 15 mM for nucleoside (4.2 mg/ml, 15 mM for isoG)and phosphate-containing molecules with equal molar amount (5.4 mg/ml, 15 mM for F-araAMP) were weighed and dissolved in double steaming water. The solution could be completely dissolved after ultrasound. The mixture was heated to 60 °C and then cooled in ice bath quickly. The reaction solution was centrifuged at 12000 rmp for 60 min, and the supernatant was taken and precipitated to remove the unreacted molecules. After overnight freezing at −20 °C and vacuum lyophilization for 12 h, the powder samples after reaction are obtained.

For G and A with phosphate-containing molecules:nucleoside molecules and phosphate-containing molecules with equal molar amount were weighed and dissolved in double steaming water. Then adding hydrochloric acid with a pH of 6 to the mixed solution to promote its dissolution. Centrifuging to remove undissolved G or A, and take the supernatant to prepare nanoparticles according to the isoG and phosphate-containing molecules process.

For isoG and nucleic acid: isoG (500 μM) and nucleic acid (100 μM) were dissolved in RNase-free water. The solution could be completely dissolved after ultrasound. The mixture was heated to 60 °C and then cooled in ice bath quickly. The reaction solution was centrifuged at 12000 rmp for 60 min, and the supernatant was taken and precipitated to remove the unreacted molecules. After overnight freezing at −20 °C and vacuum lyophilization for 12 h, the powder samples after reaction are obtained.

The sequence of siRNA and oligo nucleoside

siYAP:

(sense) UCUUUGACCAGAAGAUGUCUU;

(anti-sense) GACAUCUUCUGGUCAAAGAUA.

ASO YAP(anti-sense):

GACAUCUUCUGGUCAAAGAUA.

ASO TAZ(anti-sense)

UUCAUGUACUUGGUCCAGAAGCAGCUG.

### 2.3 Density functional theory (DFT) calculation

By molecular surface analysis, the electrostatic potential correlation is defined with the electron density profile of 0.001 a.u. The electronic structure is calculated using the DFT method of B3LYP/6-311G base group. The electrostatic potential related descriptors are calculated and visualized using Multiwfn and VMD software.

### 2.4 Molecular dynamic (MD)

The molecular dynamics simulation was performed using GROMACS software. Select AMBER14SB.ff force field and generate small molecule ligand topology file using ACPYPE software and GAFF force field. A solvent box of 10*10*10 nm with the water model as TIP3P was established. packmol software was used to randomly distribute 30 of each of the two molecules in the solvent. Na^+^/Cl^−^ is added to the system to balance the system charge. Then, the energy is minimized using the steepest descent method of 2500 steps and the conjugate gradient method of 2500 steps. At a temperature of 298.15K, 100 ps NVT ensemble simulation and 100 ps NPT equilibrium simulation were performed. Finally, under the periodic boundary condition, the dynamics simulation of 200 nm was carried out, and the PME method was used to calculate the long-range electrostatic interaction. The non-bond truncation distance was set to 1.2 nm, the collision frequency was set to 2 ps, the system pressure was set to 1 bar, the integral step was 2 s, and the trajectory was saved every 100 ps. In this system, isoG exists in the form of protonation of -NH2 to NH3^+^ and deprotonation of PPA phosphate groups.

### 2.5 In vitro drug release

A total of 2 mL of F-araAMP-isoGwas transferred to a dialysis bag (MWCO = 3.5 kDa) and then immersed in 250 mL of phosphate buffer (pH 7.4) and acetate buffer (pH 5.0) solutions with gentle shaking (100 rpm) at 37 °C in a laboratory shaker. At predetermined time intervals, 3 mL of external buffer solution was withdrawn and replaced with 3 mL of phosphate buffer or acetate buffer. The amount of released F-araAMP are detected by HPLC. The chromatography was performed on ACQUITY UPLC BEH C18(1.7 μm, 2.1 × 100 mm, Waters, Milford, MA, USA) column with mobile phases of 0.1% formic acid water (A) and acetonitrile (B) with gradient elution at a constant temperature of 35 °C. The optimized gradient elution flow rate was 0.5 mL/min, and the gradient program was: starting at 2% B, increasing linearly to 5% B for 1 min, then increasing to 90% B for 0.5 min. The gradient is then maintained at 90% B for 1.1 min. The sample size was 10 μL.

### 2.6 Preparation of Cy5 loaded F-araAMP-isoG NSN

Firstly, Cy5 was labeled onto the F-araAMP through amination reaction of carboxyl groups and amino groups. The reaction ratio of the two was 1:10 mol in sodium bicarbonate with a pH of 8.5. After stirring for 12 h, the solution was dialyzed against deionized water for 12 h (MWCO = 1 kDa) and the NaHCO_3_ was renewed every 4 h to remove the unreacted Cy5. Finally, the Cy5-loaded F-araAMPwas obtained. Cy5-loaded F-araAMP are prepared with the same procedure. The successful reaction was confirmed by high-resolution mass spectrometry. By reacting Cy5 loaded F-araAMP with isoG under self-assembly conditions, F-araAMP-isoG NSN can be obtained.

### 2.7 Cellular uptake of F-araAMP-isoG NSN

CAL-27 cells were cultured in DMEM supplemented with10% FBS and 5% antibiotics at 37 °C in a humidified atmosphere containing 5% CO2.

CLSM: CAL-27 cells were spread into a 24-well plate with crawling tablets in 2 × 10^4^ cells per cell. After the cells were affixed to the wall, they were treated with 1 μM Cy5-labeled NSN for different times (0 h, 0.5 h, 2 h, 6 h), washed with PBS 3 times, fixed with paraformaldehyde for 10 min, and then nuclear staining was performed with DAIP. Scanning is performed after sealing the tablet. When detecting the co-localization of Nano and lysosomes, the cell culture medium containing NSN was removed after treatment with Cy5-labeled NSN for 6 h. Green lysosome fluorescent probe with 75 nm concentration was incubated at 37 °C for 1 h. After the lysosomal fluorescence probe solution was removed, fixation, DAIP staining and sealing were performed, and image acquisition was performed by confocal laser scanning microscope (FV3000, Olympus, Japan).

Flow cytometry: CAL-27 cells were treated with 1 × 10^5^ cells/empty homogeneous to 6-well plates, Cy5-labeled NSN in accordance with the above concentration and time conditions, washed twice with PBS, digested by pancreatic enzymes, and centrifuged to obtain cell precipitation. After the cells were suspended with 500 μL PBS, flow detection was performed, and the channel was Cy5 fluorescence channel.

### 2.8 Apoptosis and cell cycle analysis with flow cytometry

HSC-3 and CAL-27 cells were evenly spread into 6-well plates according to 1 × 10^5^ cells per well. After the cells were attached to the wall, the cells were treated for 48 h according to experimental conditions. For apoptosis analysis, the cells were digested and collected by pancreatic enzymes and treated with Annexin V/PI apoptosis detection Kit (Key GEN, China). For cell cycle analysis, cells were treated with Apoptosis Analysis Kit (Beyotime, China). Finally, the cells were analyzed by flow cytometry (Beckman FC500, Brea, CA, USA). The data analysis was performed with FlowJo V10 software.

### 2.9 Pharmacokinetics analysis of F-araAMP-isoG NSN

Collection of blood samples: SPF BALB/nude mice (female), 20 ± 5 g, 4 weeks old. The animals were kept under standard conditions (22 ± 2 °C; 50∼70% relative humidity; 12 h alternating light). The mice were randomly divided into two groups: Group A: F-araAMP (15 mg/kg) by tail vein injection; Group B: F-araAMP-isoG NSN (15 mg/kg) were injected into the tail vein, and 0.15 mL of blood was collected from the eyeball and placed into the heparin anticoagulant tube at 5 min, 15 min, 30 min, 1 h, 2 h, 4 h, 6 h, 8 h, 24 h, 48 h and 72 h after injection, respectively. The blood sample was immediately centrifuged at 4 °C at 4000×g for 15 min and plasma was obtained.

Collection of stool and urine samples: Mice were placed in a metabolic cage after administration, and urine and feces were collected within 96 h (at 24h intervals).

Sample investigation: The samples were detected by UHPLC-MS/MS. The chromatography was performed on ACQUITY UPLC BEH C18(1.7 μm, 2.1 × 100 mm, Waters, Milford, MA, USA) column with mobile phases of 0.1% formic acid water (A) and acetonitrile (B) with gradient elution at a constant temperature of 35 °C. The optimized gradient elution flow rate was 0.5 mL/min, and the gradient program was: starting at 2% B, increasing linearly to 5% B for 1 min, then increasing to 90% B for 0.5 min. The gradient is then maintained at 90% B for 1.1 min. The sample size was 10 μL. Mass spectrum conditions: The ion source is an electrospray ionization source, the ion spray voltage is 5500 V, the temperature is 500 °C, the ion source gas 1 and 2 are 60 psi, and the declustering voltage is 100 V. IsoG and F-araAMP were detected using MRM mode, and the ion pairs were 284.20 (parent ion) → 152.10(daughter ion) and 265.2 (parent ion) ∼232.2(daughter ion), respectively. Manipulation, acquisition and data analysis using Masslynx V4.1 software. The pharmacokinetic parameters of the non-atrioventricular model were calculated by DAS2.0(Drug and Statistics for Windows) software.

### 2.10 In vivo imaging in the CAL-27 xenografts model

CAL-27 CDX model: 100 μL of CAL-27 cell suspension (2.0 × 10^6^ cells) was implanted under the right rib of female nude mice (about 20 g weight), and the tumor grew to 100 mm^3^ size about 2 weeks later, which was a successful establishment of the CAL-27 CDX model. The mice were randomly divided into three groups (n = 3). Then tumor-bearing mice were given an intravenous injection via the tail vein, Cy5-loaded F-araAMP or Cy5-loaded F-araAMP-isoG NSN at the same Cy5 concentration. The mice were anaesthetized by intraperitoneal injection of chloral hydrate, and scanned at 0 h, 0.5 h, 2 h, 6 h, 24 h and 48h using imaging system (Raycision, Chaina). All animal experiments were approved by the Ethics Committee of West China Stomatological Hospital, and all experimental animal studies followed the experimental protocol approved by the Animal Care and Use Committee of Sichuan University.

### 2.11 Hemolysis experiment

4% rabbit cell suspension, 800 μL H_2_O+200 μL red blood cell suspension as positive control, 800 μL PBS+200 μL red blood cell suspension as negative control, different concentrations of Nano solution 800 μL+200 μL red blood cell suspension as experimental group. After incubation at 37 °C for 3 h and centrifugation at 12000 rmp for 10 min, photographs were taken. The supernatant of 100 μL was placed in a 96-well plate with 3 multiple holes, and the absorbance was measured at 577 nm. Hemolysis rate (%) = (Ab sample -Ab negative)/(Ab positive -Ab negative) ×100%.

### 2.12 In vivo antitumor activity

CAL-27 CDX model experiment: The subcutaneous HSC-3 and CAL-27 CDX model was successfully established by implanting 100 μL of and CAL-27 cell suspension (2.0*107 cells/mL) at the right flank in female nude mice (around 20 g body weight). Subsequently, female nude mice bearing CAL-27 tumors with a volume of about 100 mm^3^ respectively were randomly divided into four groups (n = 5) and treated with: H_2_O (Control), F-araAMP(15 mg/kg), F-araAMP-isoG mixture(15 mg/kg) and F-araAMP-isoG NSN (15 mg/kg), ASO-NSN(1 μM/kg). They administrated once three days in the volume of 50 μL via tail vein injection. Tumor growth and mouse body weight were monitored every two days. Tumor volume was calculated by the following formula: TV (mm^3^) = a (length) *b^2^ (width)/2.

OSCC PDX model experiment: The OSCC PDX model was established by directly implanting fragments from a patient's buccal and tongue SCC tissue into a nude mouse (4-5 weeks). Briefly, surgically resected human buccal and tongue SCC tissue was obtained and sliced into small fragments (2 mm in size) to implant subcutaneously into the right flanks of the mice as the first generation of tumor (F1). After successfully establishing xenografts, they are extracted from donor mice and reimplanted into other recipient mice for further in vivo passaging. Following that, each generation is marked as second (F2), third (F3), and generation (Fn). We started therapy with the fifth generation of the PDX model in the tongue SCC PDX model research. When the tumor volume reached approximately 100 mm^3^, the mice were randomly divided into four groups (n = 5 or n = 4). The mice were treated with as mentioned above in the CAL-27 CDX model. The tumor tissues and organs were extracted from mice for histological analysis at the endpoint of the therapeutic study.

### 2.13 Histopathology analysis

The tumors and major organs were fixed with 4% formaldehyde, embedded in paraffin, and then cut into slices with 4 μm thickness. Hematoxylin and eosin (H&E, Sigma) was used to stain the fixed slices. The antibodies used in Immunohistochemistry (IHC) were as follows: Ki67 (Abcam, 1:200), Cleaved-Caspase-3 (CST,1:200), γ-H2AX (CST,1:200). TUNEL assay with an In Situ Cell Death Detection Kit according to the manufacturer's protocol (Abbkine, China). The stained tumor slices were observed with slide scanner (VS200, Olympus, Japan)

### 2.14 Statistics analysis

All the statistical analysis and graphs were performed and generated using Prism (GraphPad). The obtained data were represented as mean value ± S.E.M. Statistical differences among three or more groups were analyzed by one- or two-way analysis of variance (ANOVA) followed by Tukey's multiple comparisons post hoc test, which were considered statistically significant when *p* < 0.05 (* for *p* < 0.05, ** for *p* < 0.01, and *** for *p* < 0.001)

## 3 Results

### 3.1 Identification of 5′-Phosphate hydrogen bonding as a directional supramolecular recognition motif

**Fig. 1:**
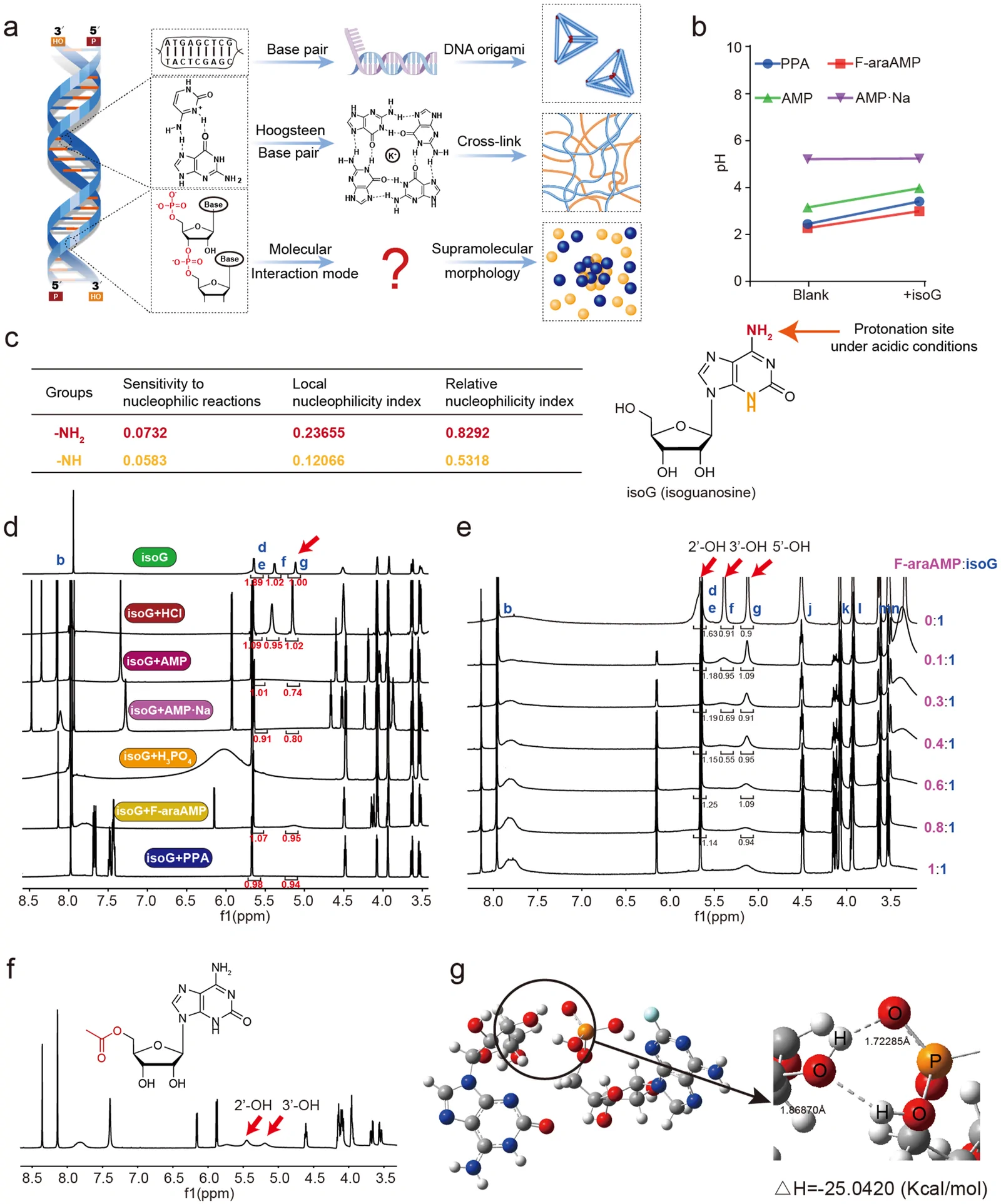
**Molecular recognition mechanism between nucleosides and phosphate groups. (a)** Conceptual illustration of the design principle. The supramolecular self-assembly strategy is inspired by the natural nucleotide architecture, where hydrogen bonding between the ribose 5′-OH and phosphate groups serves as a molecular recognition motif. **(b)** The pH change of the solution after the reaction of isoG with phosphate molecules. **(c)** Calculation of the nucleophilic index of the -NH and -NH2 of isoG. **(d)**^1^H NMR spectra of isoG alone and in mixtures with different phosphate-containing molecules in dimethyl sulfoxide *d6* solvent. **(e)**^1^H NMR spectra of isoG (15 mM) with titrated with increasing molar ratios of F-araAMP. **(f)**^1^H NMR spectra of isoG 5′-OH with acetylation modification with F-araAMP. **(g)** Binding free energy of isoG 5′-OH and F-araAMP phosphate group binding sites.

Inspired by the remarkable self-assembly behavior of DNA, we realized that although base pairing interactions have been extensively characterized, alternative molecular interaction mechanisms within the nucleotide framework remain largely underexplored, particularly those involving sugar and phosphate moieties. We hypothesize that specific molecular recognition between nucleosides and phosphate groups may drive supramolecular organization in aqueous environments, providing a new conceptual basis for nucleoside guided self-assembly (Fig. 1a). To test this hypothesis, we firstly confirmed the molecular recognition between nucleosides and phosphate containing molecules. On account of the superior assembly propensity of isoguanosine (isoG), an isomer of guanosine (G) with a neutral pKa (∼8.25). We firstly chose isoG to clarify its interaction with phosphate containing molecules. For phosphate containing molecules, adenosine monophosphate (AMP) was chosen as a canonical nucleotide bearing a phosphate group, while its sodium salt form (AMP·Na) served to assess the effect of deprotonation state. F-araAMP, a clinically approved nucleoside analog bearing chemical modifications, was included to evaluate the relevance of this interaction in drug-like molecules. Phenylphosphonic acid (PPA), which lacks a nucleobase, was selected to rule out base pairing and assess the scope of this phosphate-driven assembly. Firstly, isoG exhibited significant changes in solubility upon mixing with phosphate containing compounds in a 1:1 M ratio, even without pH adjustment (Fig. S1a). pH measurements revealed that mixing isoG with acidic phosphate solutions resulted in a measurable increase in pH (initial pH < isoG pKa = 8.25 ± 0.4), consistent with partial protonation of isoG and subsequent electrostatic interactions with deprotonated phosphate species (Fig. 1b). Nucleophilicity calculations further confirmed that isoG's -NH_2_ group participates in proton exchange under acidic conditions, enhancing its interaction with phosphate species (Fig. 1c). To localize the interaction site, we performed ^1^H NMR spectroscopy. Upon mixing isoG with phosphate containing compounds, we observed that the signal corresponding to the 5′-OH proton became significantly broadened, indicating its involvement in hydrogen bonding interactions, likely with the phosphate moiety. This broadening effect is a hallmark of dynamic hydrogen bonding interactions, where the proton engages in intermediate exchange on the NMR timescale, typically leading to signal broadening or even disappearance. The phenomenon reflects both the transient dynamics and moderate stability of the hydrogen bond formed between the 5′-OH and phosphate groups. Meanwhile, the signals for the 2′-OH and 3′-OH protons disappeared entirely, indicating potentially involvement in hydrogen bonding. Notably, nucleobase signals (-NH_2_, -NH) remained unaffected, suggesting specificity to the sugar moiety. Control experiments using HCl or H_3_PO_4_ confirmed that this interaction is phosphate-specific rather than pH-driven (Fig. 1d and Fig. S1b). To confirm the primary hydroxyl group involved in the phosphate mediated interaction, we conducted NMR titration by keeping the isoG concentration constant (15 mM) while gradually increasing the concentration of F-araAMP (Fig. 1e). The 5′-OH signal exhibited progressive broadening and eventually merged into a broad envelope, consistent with a concentration dependent dynamic interaction, most likely via hydrogen bonding. In contrast, the 2′- and 3′-OH signals disappeared immediately upon the initial addition of F-araAMP, suggesting their sensitivity to the assembly environment but not strong or direct interaction. This interpretation is supported by further analysis using a synthetically modified isoG lacking the 5′-OH group, which, upon F-araAMP addition, showed no significant spectral changes for the remaining 2′- and 3′-OH signals (Fig. 1f). These results support the involvement of the 5′-OH group in phosphate-associated hydrogen bonding, while the 2′- and 3′-OH groups may undergo non-specific solvent exchange or weak transient interactions.

To assess the effect of 5′-OH phosphorylation on neighboring groups, we compared the ^1^H NMR spectra of nucleosides and their corresponding 5′-nucleotides. The phosphorylation of 5′-OH significantly altered the chemical shifts of the adjacent 2′-OH and 3′-OH protons (Fig. S1c), indicating that non-covalent binding at the 5′-monophosphate may also influence neighboring groups through intramolecular hydrogen bonding and electronic effects. FTIR spectra show P=O and P-O-H bands (1136 cm^−1^, 1370 cm^−1^) in the F-araAMP-isoG assemblies, consistent with hydrogen bonding involving the phosphate group (Fig. S1d). DFT calculations supported these findings. Electrostatic potential surface mapping showed strong complementarity between the electron rich phosphate and electron deficient 5′-OH and -NH_2_ groups on isoG (Fig. S1e). The binding energy between isoG and F-araAMP was calculated as −25.04202162 kcal/mol, with two stable hydrogen bonds (O···H distances of 1.72285 and 1.86870 Å) (Fig. 1g and Fig. S1f).

These results suggest a potential modular assembly mechanism between isoG and phosphate containing molecules in water, which we further explore through supramolecular self-assembly studies.

### 3.2 Uncovering the self-assembly behavior of nucleosides and phosphate containing molecules in aqueous solution

**Fig. 2:**
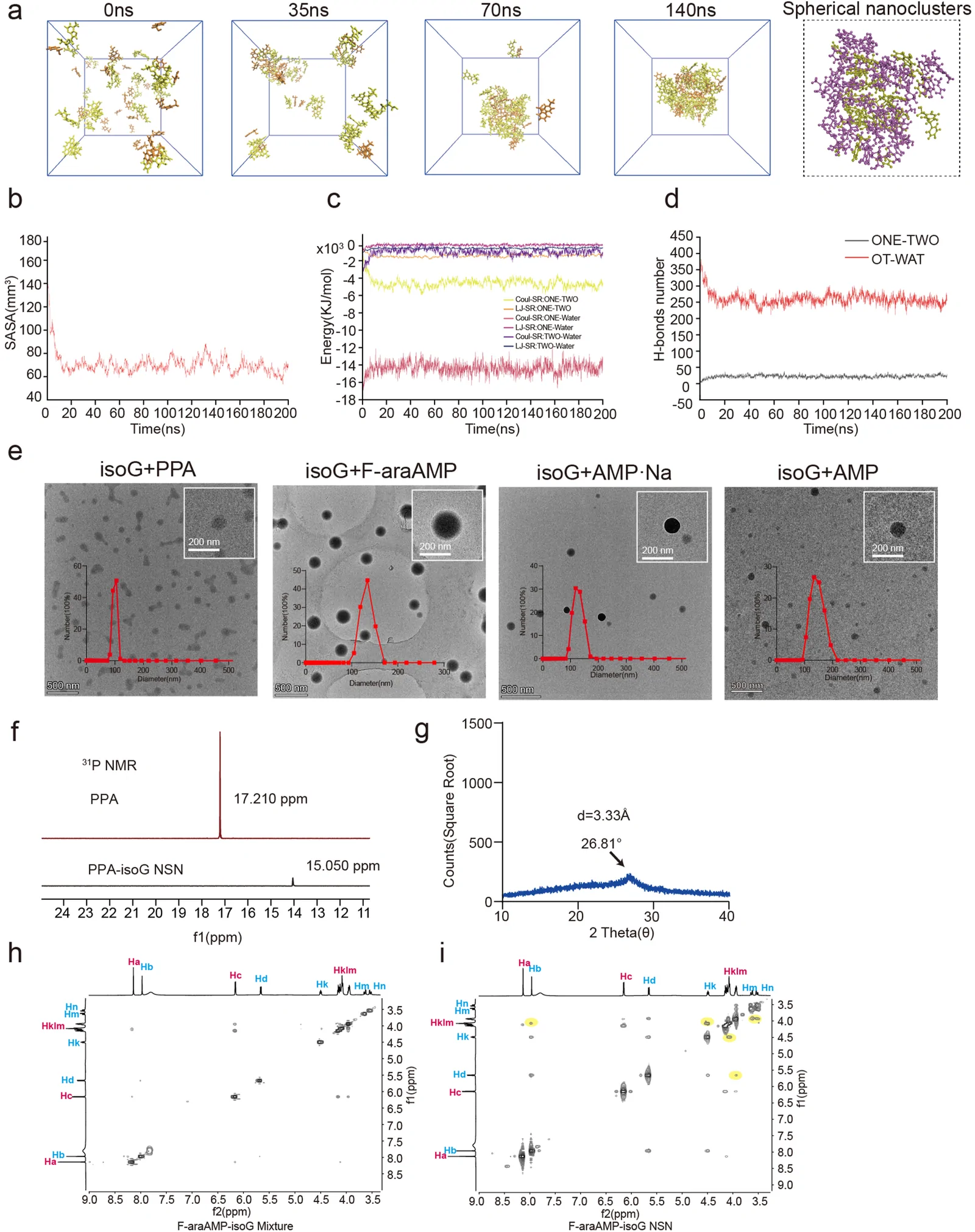
**The self-assemble process of NSN. (a)** Representative MD snapshots showing the self-assembly pathway of isoG and PPA at four time points. **(b)** SASA fraction during the self-assemble process. **(c)** Interaction energy profile of PPA-isoGduring the self-assembly process. **(d)** Temporal evolution of the number of intermolecular hydrogen bonds in PPA-isoGassemblies. **(e)** Representative TEM image and DLS showing the formation of nucleoside supramolecular nanostructures. **(f)**^31^P NMR spectra of PPA-isoG NSN. **(g)** The PXRD pattern of PPA-isoG NSN. **(h)**^1^H-^1^H NOESY spectra of F-araAMP-isoG mixture. **(i)**^1^H-^1^H NOESY spectra of F-araAMP-isoG NSN (Yellow marks indicate increased intermolecular interactions).

Amphiphilic molecules can assemble into supramolecular structures in water through hydrophobic interaction [16]. Hydrophobic nucleosides and hydrophilic phosphate containing molecules form amphiphilic molecules through electrostatic recognition and hydrogen bonds. To visualize the self-assembly mechanism at atomic resolution, we performed molecular dynamics (MD) simulations using protonated isoG and deprotonated PPA (eliminate potential interference from nucleobase) (Fig. S2a). Rapid nucleation occurred within 20 ns, reaching structurally consolidated nanoclusters by ∼140 ns (Fig. 2a). SASA analysis showed a sharp surface area decrease followed by stabilization (Fig. 2b), and radius of gyration measurements confirmed isotropic nanostructure formation (Fig. S2b). Interaction energy calculations revealed strong Coulombic (−4599 kJ/mol) and Lennard-Jones (−1422 kJ/mol) contributions (Fig. 2c), emphasizing the roles of electrostatics and van der Waals forces. However, such non-specific interactions alone do not account for the molecular selectivity or the formation of defined nanostructures. 5′-OH blocked isoG analogues failed to assemble, underscoring the necessity of site specific hydrogen bonding. Over 250 H-bonds were formed with water, forming a hydrated shell that supports aqueous dispersibility and hydrogen bond analysis during the simulation showed ∼60 stable PPA-isoG H-bonds, ∼38% involving the 5′-OH group (Fig. 2d). This behavior suggests a specific and spontaneous molecular recognition between isoG and phosphate groups, likely influenced by isoG's unique acid base characteristics. To verify the results of MD, we explored that isoG and phosphate containing molecules self-assemble via sonication in water followed by thermal cycling (heating and rapid cooling), promoting non-covalent polymerization (e.g., hydrogen bonding and electrostatic interaction) between the nucleoside and phosphate components. As shown in transmission electron microscopy (TEM) and DLS results, isoG can assembled with various phosphate containing molecules to form spherical nanoparticles (Fig. 2e). It's worth to note that AMP·Na, AMP, F-araAMP all contain base pair, which may contribute to assembly through canonical base pairing interactions. As the MD results showed that PPA is still able to co-assemble with isoG into well defined nanostructures under the same conditions. This observation suggests that phosphate associated interactions, including 5′-OH phosphate hydrogen bonding, contribute substantially to the assembly process, while canonical base specific recognition is unlikely to be the sole determinant.

To further verify the successful construction and characteristics of NSN.We con ducted ^31^P NMR, the spectra shift 0.363 ppm and 2.105 ppm for F-araAMP-isoG NSN and PPA-isoG NSN, respectively, confirming changes in the chemical environment of the phosphate group due to intermolecular interactions (Fig. 2f, and Fig.S2c). To investigate possible secondary structures within the nanostructures, powder X-ray diffraction (PXRD) was performed. A broad peak centered at 26.81° was observed, consistent with π-π stacking between aromatic rings (Fig. 2g). ^1^H-^1^H NOESY results further confirmed the successful assembly of F-araAMP-isoG NSN, and the enhancement of hydrogen interaction in F-araAMP-isoG NSN (Fig. 2h and i, Fig. S2d). Supporting experiments revealed a High-resolution electrospray ionization mass spectrometry (HRMS) was used to probe the stoichiometry of the assemblies. A 1:1 molecular ratio between isoG and F-araAMP was confirmed, both for isoG and *d*-labeled isoG (*d*-isoG) [17], indicating a consistent assembly pattern (Fig. S2e and f). The UV results showed the characteristic peaks both F-araAMP and isoG in F-araAMP-isoG NSN (Fig. S2g). A zeta potential of −7.25 mV, indicating colloidal stability in water (Fig. S2h). To exclude the involvement of canonical base pairing, circular dichroism (CD) spectra were recorded. No characteristic signals associated with G-quadruplexes or other nucleobase specific assemblies were observed, suggesting that base pairing interactions do not contribute to the assembly process (Fig. S2i).

Taken together, these results show that isoG, as a core nucleoside, can assemble with a series of phosphate containing molecules to form supramolecular nanoparticles. We further explored the programmable of NSN and its potential applicability to other nucleosides.

### 3.3 The adjustability and broadly applicable of NSN

**Fig. 3:**
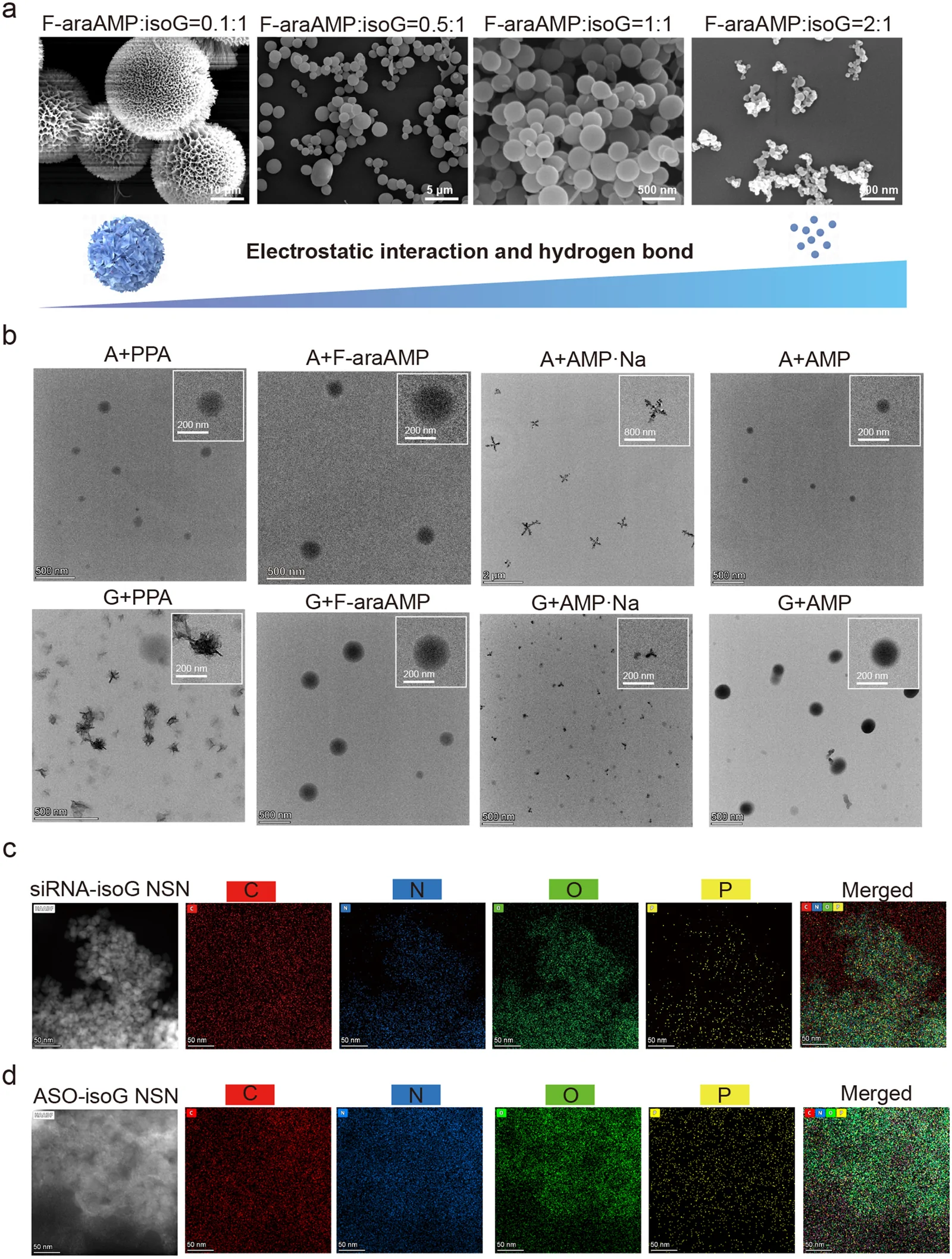
**Self-assembly of nucleosides with phosphate-containing molecules in water. (a)** The SEM spectra of F-araAMP-isoG NSN morphology under different ration**. (b)** Representative TEM image of nucleoside supramolecular nanostructures. **(c)** EDS spectra of siRNA-isoG NSN. **(d)** EDS spectra of ASO-isoG NSN.

As shown in Fig. 1b, the hydroxyl peak gradually changes with increasing concentration of F-araAMP, indicating the enhancement of non-covalent interaction between the isoG and F-araAMP. We further verified the influence of the reaction ratio on the assembly morphology. At low F-araAMP concentrations, micro-flower formed. As the F-araAMP concentration increased, more compact spherical NPs emerged, reaching nanoscale dimensions at a 1:1 ratio (Fig. 3a). These results suggest that isoG and F-araAMP spontaneously co-assemble into nanoscale supramolecular structures under aqueous conditions, likely stabilized by cooperative non-covalent interactions, including 5′-OH phosphate hydrogen bonding, electrostatic interactions, and nucleobase stacking. The system is tunable through rational pairing of nucleoside units with phosphate containing counterparts, allowing the programmable formation of nanostructures in fully aqueous media.

To verify the broadly applicable of this assembly mechanism, we replaced isoG with guanosine (G) and adenosine (A), along with a set of representative phosphate containing compounds to explore their supramolecular assembly behavior. Initial attempts to co-assemble G or A with phosphate based molecules under neutral conditions showed limited interaction and solubility (Fig. S3a). Since no significant self-assembly was observed under neutral conditions due to poor solubility, indicating that the protonation state of nucleosides might influence their interaction with phosphate groups. To investigate this, we acidified the solution by introducing exogenous H^+^, which enhanced solubility and appeared to promote intermolecular interactions. Subsequent thermal treatment (heating and ice bath cooling) induced the formation of nanostructures. As the properties of isoG, G and A can also assemble with nucleoside monophosphates (AMP·Na, GMP, AMP), non-nucleosidic phosphate ester (phenyl phosphate, PPA), and nucleoside analog drug (F-araAMP) under 1:1 ratio. TEM revealed that these nucleoside phosphate assemblies exhibited diverse morphologies, such as spherical and flower-like nanostructures (Fig. 3b), suggesting a universal self-assembly behavior. In addition to adenine nucleoside, we further verified the self-assembly ability of pyrimidine nucleoside and deoxynucleoside with phosphate containing molecules. We found that cytidine (C), uridine (U) and thymidine (T) can also self-assemble with F-araAMP (Fig. S3b). Since the assembly of nucleosides are independent on sugar, 2′-deoxyguanosine (dG), 2′-deoxyadenosine (dA), and 2′-deoxycytidine (dC) can also effectively self-assemble with F-araAMP (Fig. S3c). The verification of this in various nucleosides further demonstrates the broad applicability of this assembly principle.

We further examined whether the NSN strategy could be extended from small molecule nucleoside analogues to biological macromolecules. Remarkably, isoG co-assembled with short RNA and oligonucleotides RNA into nanoparticles with uniform and well defined morphologies, as observed by TEM. Elemental analysis using EDS confirmed the uniform presence of C, N, O, and P within the assembled NPs, supporting successful incorporation of nucleic acid (Fig. 3c and d).

Together, these data define a dual interaction model (Fig. S3d): electrostatic forces enable initial association, while 5′-OH-phosphate hydrogen bonds impart directional molecular recognition, facilitating stable nanostructure formation in water. This 5′-OH-phosphate hydrogen bond adds to the repertoire of supramolecular interactions available for programmable nucleoside assembly in aqueous environments.

### 3.4 Self delivery of therapeutic nucleoside analogues via NSN

Phosphate modification is a common strategy in drug design to improve solubility, stability, and bioavailability, such as F-araAMP. Although F-araAMP has been widely used in the treatment of hematologic malignancies, its therapeutic efficacy in solid tumors remains unsatisfactory in vivo, despite showing promising cytotoxicity in vitro [18,19]. To address this challenge, we explored whether co-assembly with isoG under mild aqueous conditions could improve F-araAMP's pharmacological profile by forming stable nanostructures (Fig. S4a).

**Fig. 4:**
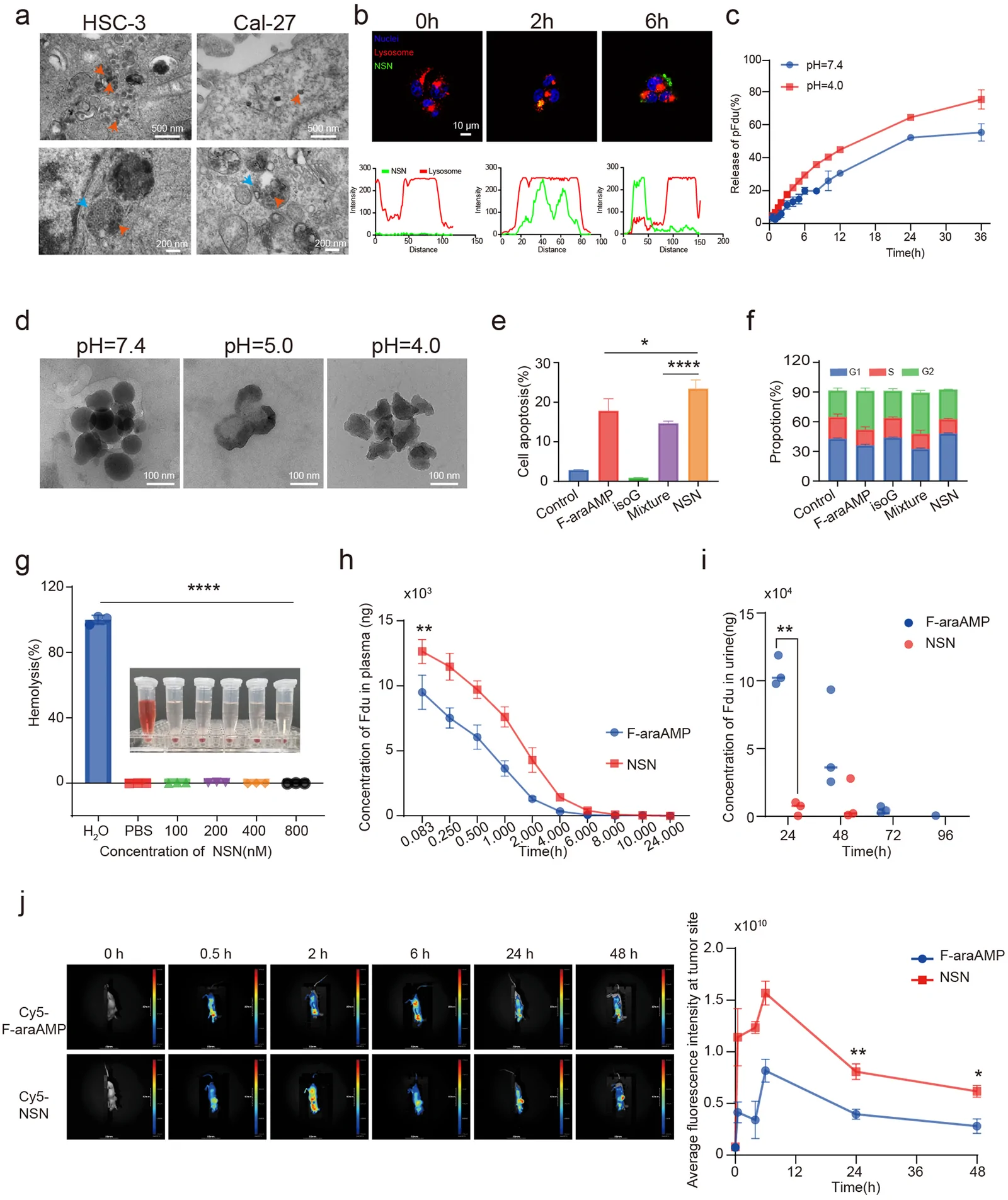
**Cellular uptake and pharmacokinetics of F-araAMP-isoG NSN. (a)** Representative TEM image of OSCC cells after treatment with F-araAMP-isoG NSN. (orange arrow: F-araAMP-isoG NSN; bule arrow: lysosome) **(b)** Confocal fluorescence microscopy images showing lysosome escape of Cy5-labeled F-araAMP-isoG NSN. **(c)** In vitro release kinetics of F-araAMP from F-araAMP-isoG NSN under different pH conditions (pH 4.0 and 7.4). **(d)** Morphological evolution of F-araAMP-isoG NSN at different pH values. **(e)** Flow cytometry analysis of apoptosis in CAL-27 cells treated with free F-araAMP, isoG, F-araAMP-isoGmixture, and F-araAMP-isoG NSN (300 nM, 48 h, n = 3). **(f)** Cell cycle distribution of CAL-27 cells under the same treatment conditions (n = 3). **(g)** Hemolysis assay of F-araAMP-isoG NSN (n = 3). **(h)** Plasma concentration–time profiles of free F-araAMP versus F-araAMP-isoG NSN following intravenous administration in mouse (n = 3). **(i)** Cumulative urinary excretion of Fdu after intravenous administration of free F-araAMP or F-araAMP-isoG NSN in mouse (n = 3). **(j)** In vivo NIRF whole-body imaging and tumor-site fluorescence quantification in CAL-27 CDX models after intravenous injection of free Cy5, Cy5-loaded F-araAMP, or Cy5-loaded F-araAMP-isoG NSN (n = 3).

Building upon the physicochemical characterization of F-araAMP-isoG NSN, we next evaluated their biological performance in vitro and in vivo. As previously noted, F-araAMP shows promising antitumor activity in vitro but suffers from poor pharmacokinetics and limited efficacy in vivo, particularly in solid tumors. We hypothesized that the F-araAMP-isoG NSN could enhance its intracellular delivery, improve drug stability, and enable controlled release under physiological conditions. To assess cellular uptake, Cy5-labeled F-araAMP-isoG NSN were incubated with CAL-27 (A solid tumor cell line origin from tongue). Fluorescence microscopy revealed time-dependent internalization, with progressive accumulation of Cy5 signal in the cytoplasm, indicating efficient cellular entry of the NSN (Fig. S4b–d). TEM further confirmed the presence of NSN within intracellular compartments (Fig. 4a). To explore intracellular trafficking, co-localization studies using lysosomal trackers demonstrated that the NSN localized to lysosomes while later escaping from lysosome(Fig. 4b). Given the acidic environment of lysosomes and the pH-sensitive nature of hydrogen bonded assemblies, we evaluated the pH-responsive drug release profile. In vitro release assays showed that F-araAMP was released more rapidly at pH 4.0 than at physiological pH 7.4 (Fig. 4c), consistent with a triggered release mechanism. Structural characterization also revealed gradual disintegration of the supramolecular assembly under acidic conditions (Fig. 4d), further supporting pH-dependent destabilization.

To determine therapeutic efficacy, we assessed the cytotoxicity of F-araAMP-isoG NSN in CAL-27 cells. Both the NSN and free F-araAMP exhibited dose dependent inhibition of cell viability, and the NSN exert better pro-apoptosis potency (Fig. 4e and Fig. S4e). Flow cytometry analysis revealed G1-phase cell cycle arrest following NSN treatment (Fig. 4f and Fig. S4f), consistent with the known mechanism of F-araAMP. Importantly, the NSN demonstrated excellent colloidal stability over fifteen days with minimal changes in hydrodynamic diameter in H_2_O and normal saline (Fig. S4g), supporting its potential for long circulating behavior. Encouraged by these in vitro results, we further evaluated the in vivo pharmacokinetics and biodistribution of the NSN. Hemolysis assays indicated excellent blood compatibility (Fig. 4g). Pharmacokinetic analysis revealed a significantly higher plasma AUC for the NSN group compared to free F-araAMP and isoG alone (Fig. 4h, Fig. S4h), suggesting prolonged systemic exposure. Metabolite profiling indicated that while approximately one third of free F-araAMP was excreted as fludarabine (Fdu) in urine, the NSN group showed minimal Fdu accumulation (Fig. 4i), suggesting reduced renal clearance and improved metabolic stability. Finally, in vivo fluorescence imaging demonstrated that Cy5-labeled NSN accumulated more efficiently in tumors than free F-araAMP, showing stronger and more sustained fluorescence signals that peaked at 6 h post injection, consistent with the enhanced permeability and retention (EPR) effect (Fig. 4j).

These results demonstrate that the F-araAMP-isoG NSN self delivery system enables efficient cellular uptake, pH-responsive drug release, improved pharmacokinetic behavior, and enhanced tumor accumulation.

### 3.5 Therapeutic efficacy and biosafety of the NSN in CDX and PDX tumor model

Next, we evaluated the therapeutic efficacy of the F-araAMP-isoG NSN in vivo using multiple tumor models. To comprehensively assess performance across tumor heterogeneity, we employed three xenograft models: a cell line-derived xenograft (CDX) using CAL-27 cells, and two patient-derived xenograft (PDX) models originating from tongue and buccal squamous cell carcinomas, respectively.

**Fig. 5:**
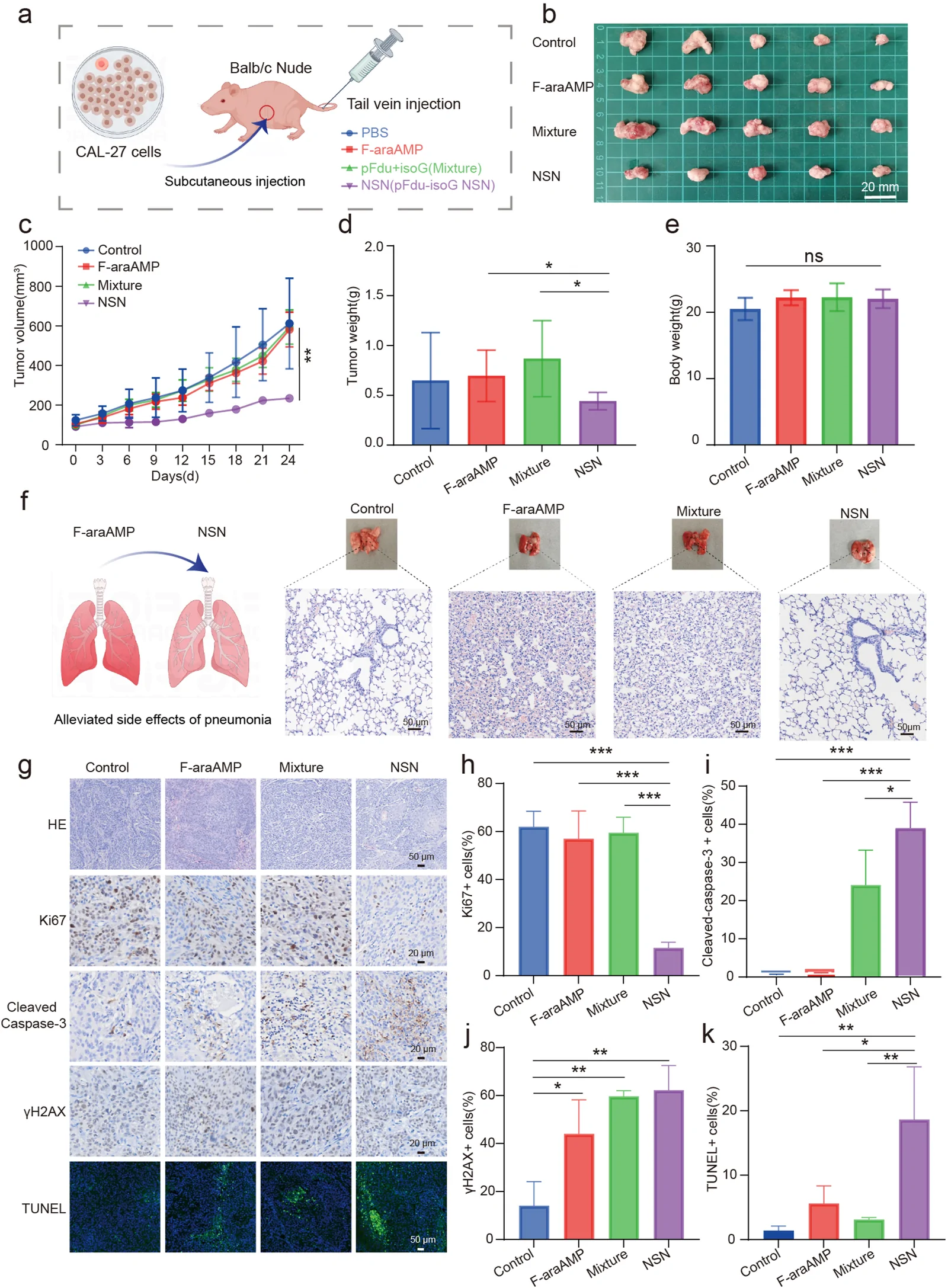
**Therapeutic efficacy of F-araAMP-isoG NSN in oral cancer CDX models. (a)** Schematic of experimental groups and treatment regimen for CAL-27 CDX model. **(b)** Images of CAL-27 CDX at the end of the experiment. **(c)** Tumor growth curves CAL-27 CDX mice (n = 5). **(d)** Tumor weight of CAL-27 CDX at the end of the experiment (n = 5). **(e)** Body weight of CAL-27 CDX mouse at the end of the experiment (n = 5). **(f)** Representative lung images and H&E staining images showing pulmonary toxicity after different treatments. **(g)** Representative HE and IHC staining images of CAL-27 CDX tumor after different treatment. **(h)** IHC analysis of Ki67(n = 3). **(i)** IHC analysis of cleaved caspase-3 (n = 3). **(j)** IHC analysis of cleaved γH2AX. **(k)** IHC analysis of TUNEL (n = 3).

We firstly constructed the CAL-27 CDX tumor model and conducted group administration (Fig. 5a). Results indicated that treatment with F-araAMP-isoG NSN led to significantly increased tumor growth inhibition compared to both free F-araAMP and a simple mixture of isoG and F-araAMP (Fig. 5b–d), tumor inhibition rates reached 60.13%. Statistical analysis confirmed the superiority of the NSN group compard with mixture group: *p* < 0.01. Importantly, no significant body weight loss was observed across any treatment group, suggesting good systemic tolerability (Fig. 5e). Interestingly, histopathological analysis of lung tissue via H&E staining revealed signs of pulmonary toxicity, including hemorrhage, inflammatory infiltration, and alveolar disruption, in mice treated with free F-araAMP or F-araAMP-isoG mixture. In contrast, mice receiving the NSN showed normal lung architecture comparable to untreated controls, indicating that the supramolecular formulation substantially reduced off-target toxicity, particularly in the lungs (Fig. 5f). HE staining of the remaining important organs showed no obvious toxicity, further confirming the safety of NSN (Fig. S5a). To explore the underlying biological processes, we performed IHC staining on tumor tissues. The NSN-treated tumors displayed markedly reduced Ki67^+^ proliferative cells, increased cleaved caspase-3^+^ apoptotic markers, elevated γ-H2AX^+^ DNA damage signals, and TUNEL assay indicating effective induction of apoptosis (Fig. 5g–k).

**Fig. 6:**
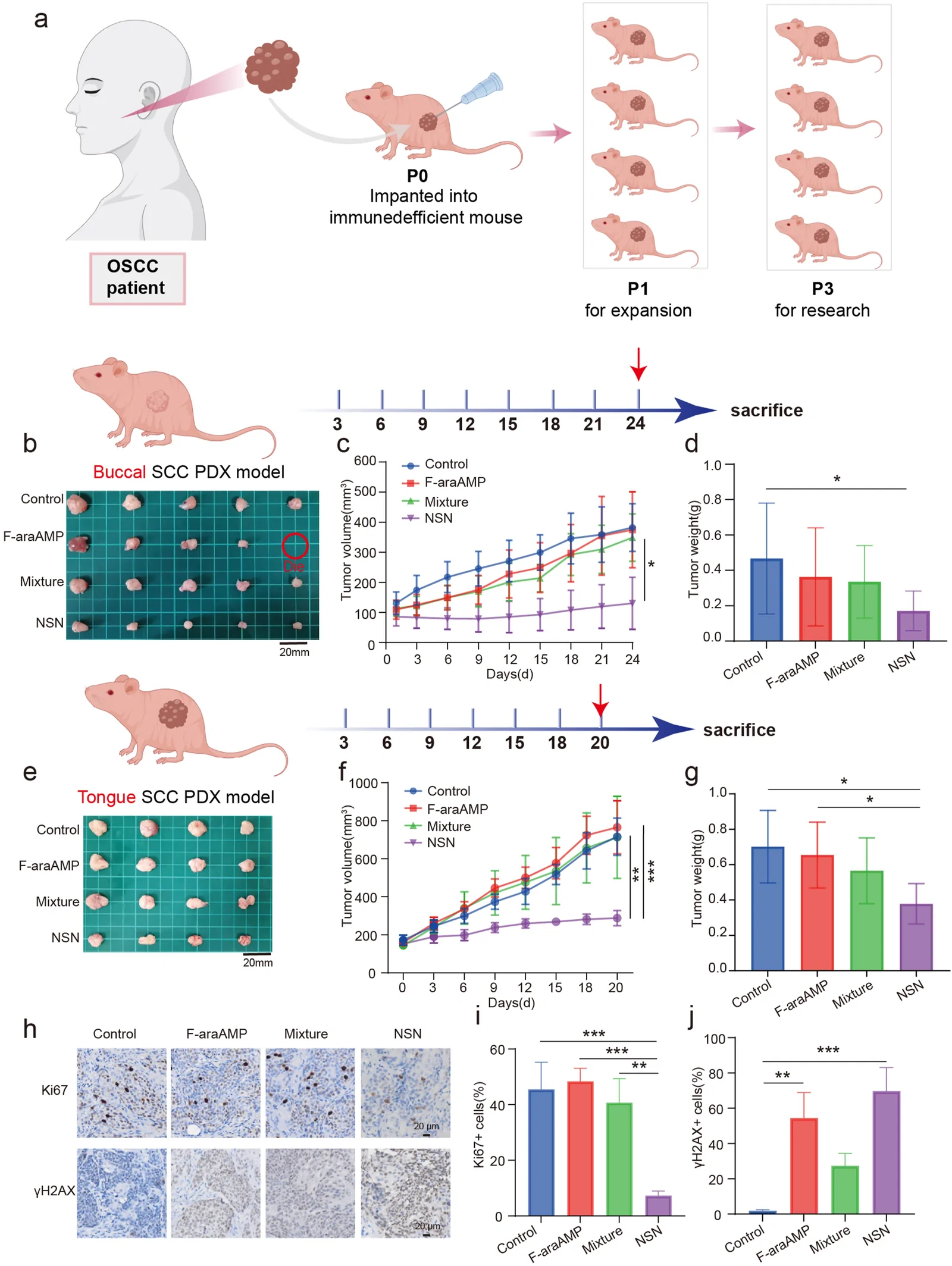
**Therapeutic efficacy of F-araAMP-isoG NSN in oral cancer PDX models. (a)** Schematic of PDX modeling **(b)** Images of buccal SCC PDX at the end of the experiment. **(c)** Tumor growth curves of buccal SCC PDX mice under different treatments (n = 5). **(d)** Tumor weight of SCC PDX mice at the end of the experiment (n = 5). **(e)** Images of tongue SCC PDX at the end of the experiment (n = 4). **(f)** Tumor growth curves of tongue SCC PDX mice under different treatments (n = 4). **(g)** Tumor weight of tongue SCC PDX mice at the end of the experiment (n = 4). **(h)** Representative IHC staining images of buccal PDX tumor after different treatment. **(i)** IHC analysis of Ki67 (n = 3). **(j)** IHC analysis of cleaved γH2AX (n = 3).

As the PDX model can reflect the physiological characteristics and microenvironment of tumors and predict treatment responsiveness, we further collected buccal SCC and tongue SCC tissues to construct PDX model to confirm the therapeutic effect of F-araAMP-isoG NSN more preciously (Fig. 6a). For buccal SCC model treatment, F-araAMP-isoG NSN showed 69.00% inhibition rate and *p* < 0.05 compared with mixture group, tumor weight was also significantly lower than control group (Fig. 6b–d). The same treatment effect are further confirmed by tongue SCC PDX. As the F-araAMP-isoG achieved 59.80% inhibition rate and *p* < 0.01 compared with mixture group, the tumor weight was also downregulated (Fig. 6e–g). The body weight of the mice did not decrease during the administration process, indicating the safety (Fig. S5b and 5c). For IHC anlysis, NSN treatment group showed more proliferation inhibition, DNA damage and pro-apopopsis effect for buccal SCC models (Fig. 6h–j, Fig.S5d and S5e).

Together, these findings demonstrate that the F-araAMP-isoG NSN not only enhances anti-tumor efficacy across multiple oral cancer models but also mitigates F-araAMP-induced systemic toxicity. This non-covalent, hydrogen bond based NSN system offers a promising platform for the clinical translation of nucleotide analog based chemotherapeutics in solid tumors.

### 3.6 Self-delivery of biological macromolecules via NSN

**Fig. 7:**
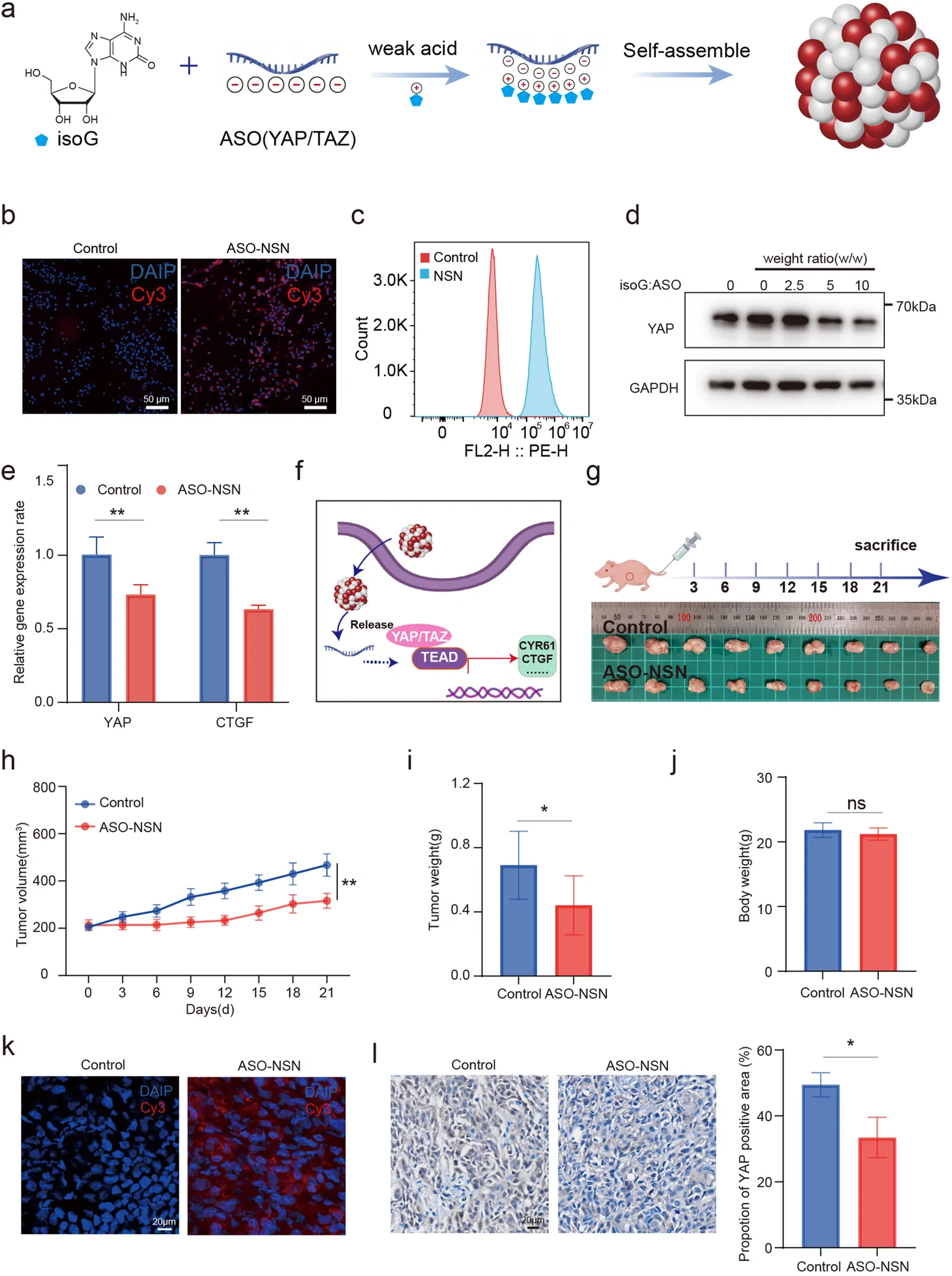
**Therapeutic efficacy of ASO-isoG NSN in oral cancer models. (a)** Schematic diagram of the self-assemble of ASO-isoG NSN. **(b)** Confocal fluorescence microscopy images showing cellular uptake of Cy3-labeled ASO-isoG NSN. **(c)** Flow cytometry analysis of cellular Cy3 fluorescence intensity. **(d)** The protein expression of YAP under ASO-isoG NSN treatment was detected by WB. **(e)** The gene expression of YAP and CTGF under ASO-isoG NSN treatment detected by qPCR. **(f)** Schematic diagram of ASO-isoG releasing ASO for anti-tumor effects. **(g)** Representative tumor images, and body weight changes. **(h)** Tumor growth curves of CAL-27 CDX mice under different treatments (n = 9). **(i)** Tumor weight of CAL-27 CDX mice under different treatments (n = 9). **(j)** Body weight monitoring of CAL-27 CDX mice on day 21 (n = 9). **(k)** Fluorescence detection of cy3 in frozen tumor sections. **(l)** IHC analysis of YAP(n = 3).

Yes-associated protein (YAP; also known as YAP1) and transcriptional co-activator PDZ-binding motif (TAZ) are core effect factors of Hippo pathway. After entering the nucleus, YAP/TAZ exerts a tumor-promoting effect by facilitating the transcription of downstream target genes, such as CYR61 and CTGF [20]. However, the surface structure of the YAP/TAZ protein is smooth and it is regarded as an undruggable target. Therefore, directly inhibiting YAP/TAZ with nucleic acid exhibit significant anti-tumor significance. Based on our NSN, we considered to construct YAP/TAZ targeted antisense oligonucleoside(ASO)-isoG NSN to delivery in vivo (Fig. 7a). These ASO-isoG NSN were efficiently internalized by cells, as confirmed by confocal microscopy and intracellular fluorescence detection through flow cytometry (Fig. 7b and c). YAP was successfully silenced in vitro as the weight ratio over 5 (Fig. 7d). These results further confirmed by qPCR as the downregulation of YAP and its downstream gene CTGF (Fig. 7e). These findings highlight the functional scope of the NSN toward gene modulation applications (Fig. 7f). We next performed a preliminary in vivo proof of concept study to evaluate the biological activity of ASO-isoG NSN. We constructed CAL-27 CDX model and administered the ASO-isoG NSN through tail vein injection. After 21 days, the growth rate of tumor was significantly inhibited under ASO-isoG NSN treatment group compared with control group (Fig. 7g and h). The tumor weight was also significantly lower than that of the control group (Fig. 7i). What's more, there was no significant difference of body weight on the last day of administration, indicating the biological safety of ASO-isoG NSN (Fig. 7j). We further performed Cy3 fluorescence labeling on ASO and detected the fluorescence of tumors after ASO-isoG NSN administration. The confocal image displayed Cy3 fluorescence accoulation in the tumors, suggesting that ASO-associated fluorescence could be detected in tumor tissues after ASO-isoG NSN administration (Fig. 7k). To further verify the successful delivery of ASO within tumors, we conducted IHC staining of YAP. The results showed that the significant reduction of nuclear YAP, indicating the successful knockdown of YAP in vivo (Fig. 7l and Fig. S6a). Ki67 staining further indicated reduced tumor cell proliferation after ASO-isoG NSN treatment, supporting the preliminary biological activity of this formulation (Fig. S6a).

## 4 Discussion and conclusion

Nucleotides, the fundamental units of DNA and RNA, assemble into double helices via canonical base pairing, hydrophobic interactions, and π-π stacking [21]. These non-covalent forces are also central to a variety of supramolecular architectures based on nucleosides and nucleotides. For instance, guanosine (G) and adenosine (A) have been widely used as supramolecular building blocks due to their versatile hydrogen bonding properties [22,23]. G is especially notable for forming G-quartet structures via Hoogsteen hydrogen bonds stabilized by metal ions, providing a robust platform for designing functional hydrogels [[24], [25], [26]]. In parallel, the structural isomer isoG exhibits self-assembling ability through planar hydrogen-bonding networks involving the O2-C2-N3 plane and N6-H donors [27], enabling the construction of isoG-based hydrogels with programmable properties [28,29].

Despite these advances, prior assemblies largely depend on canonical base pairing and ion mediated stabilization. Whether nucleosides can undergo supramolecular assembly via non-classical mechanisms remains an open and intriguing question. Interestingly, guanosine monophosphate (GMP), differing from guanosine only by a phosphate group, displays markedly enhanced self-assembly capacity. These findings have led to the proposal of a G-tether concept and suggest that the phosphate group serves as a key driver of assembly [30,31]. From a physicochemical perspective, phosphate groups are highly charged and multifunctional, acting as both hydrogen bond donors and acceptors, and can engage in electrostatic, hydrogen bonding, and coordination interactions [[32], [33], [34]]. Ribose hydroxyl groups likewise participate in hydrogen bonding, suggesting a plausible but underexplored mode of interaction between nucleosides and phosphate groups in aqueous solution [34,35].

Guided by this rationale, our study supports a plausible nucleoside phosphate assembly model involving interactions between the ribose 5′-OH group and phosphate groups. Rather than being governed by a single force, this assembly process likely arises from cooperative non-covalent interactions, including electrostatic pre-organization, 5′-OH phosphate hydrogen bonding, nucleobase stacking, and solvation effects. The failure of 5′-OH blocked isoG analogues to form nanostructures further supports the importance of the 5′-OH group in this assembly process. However, because direct structural evidence such as a co-crystal structure remains unavailable, the proposed 5′-OH phosphate interaction should be regarded as the most plausible mechanistic model supported by multiple complementary techniques rather than a definitively established structural interaction.

This phosphate bridge assembly mechanism offers key advantages for translational applications. Phosphate-containing molecules, including nucleotides, siRNAs, and cyclic dinucleotides, have shown promise in cancer therapy, immunotherapy, and gene regulation. However, their clinical potential is often constrained by rapid degradation, limited bioavailability, and systemic clearance [5,36,37]. To address these challenges, we introduce a minimalistic and carrier free platform. NSN system, which utilizes the intrinsic non-covalent complementarity between nucleosides and phosphate groups to form dynamic, fully drug-loaded nanostructures in aqueous solution. This strategy avoids chemical modification or external carriers, while enhancing biological stability, circulation time, and tissue accumulation. As a proof of concept, we co-assembled isoG with the phosphate-modified anticancer drug F-araAMP into stable NSN. These supramolecular nanostructures exhibited prolonged systemic circulation, reduced renal clearance, and eliminated urinary excretion. In an oral cancer model, F-araAMP-isoG NSN significantly improved tumor accumulation and antitumor efficacy, while concurrently reducing systemic toxicity, including pulmonary inflammation. The improvement in efficacy mainly results from the enhanced in vivo circulation and promoted cellular uptake. More importantly, for ASOs, their poor cellular permeability, rapid renal clearance, and short plasma half-life are well-recognized pharmacokinetic limitations that necessitate carrier-assisted delivery for therapeutic efficacy. IsoG administrated alone in other experiments showed no therapeutic effect (Figs. 5c–6b and e). The ASO-isoG NSN showed enhanced therapeutic effect in vivo, suggesting that the established NSN system serve as a supramolecular platform for self delivering nucleoside therapeutics, enabling programmable nanostructure formation without covalent engineering. We acknowledge that the ASO-isoG NSN study was designed as an initial proof of concept experiment, and several important control groups were not included, such as free ASO, isoG alone, non-assembled ASO/isoG mixtures, and scrambled or non-targeting ASO formulations. Therefore, the current ASO data cannot rigorously define the specific contribution of the NSN delivery platform or exclude sequence independent effects. Future studies incorporating these controls will be essential to validate the delivery specific efficacy, target specificity, and therapeutic mechanism of ASO-isoG NSN.

There are some limitations for our study. First, although our NMR, FTIR, DFT, molecular dynamics simulation, and 5′-OH blocking experiments collectively support the proposed 5′-OH phosphate interaction model, direct structural evidence is still unavailable. Future studies using co-crystal structures, solid state NMR, or other high resolution structural approaches will be needed to directly define the molecular geometry of this interaction. Second, The hydrogen-bonding assignments were made possible by using DMSO-*d*_6_ to avoid H-D exchange of labile protons; this provides structural insight into the recognition geometry, though the solvent does not replicate the aqueous assembly conditions. Finally, while our in vitro uptake and apoptosis results indicate that the NSN formulation can enhance cellular internalization and biological activity, the improved in vivo antitumor efficacy is most directly supported by the favorable pharmacokinetic profile of F-araAMP-isoG NSN, including prolonged circulation, reduced systemic clearance, improved stability, and increased tumor accumulation.

In conclusion, this study proposes a robust, non-canonical molecular recognition mechanism between nucleosides and phosphate containing molecules that enables precise and efficient self-assembly in aqueous environments. This finding expands the conceptual framework of nucleotide interactions beyond classical base pairing and provides a versatile supramolecular strategy for the design of carrier-free, biologically active nanomedicines with clinical relevance.

## CRediT authorship contribution statement

**Jiamin Yao:** Conceptualization, Investigation, Methodology, Visualization, Writing – original draft. **Liu Liu:** Conceptualization, Visualization. **Junfeng Wang:** Investigation, Methodology. **Kaichao Wang:** Investigation, Validation. **Jiang Liu:** Methodology, Resources, Visualization, Writing – review & editing. **Yao Yuan:** Conceptualization, Methodology, Resources, Visualization, Writing – review & editing. **Hang Zhao:** Resources, Validation, Visualization, Writing – review & editing.

## Patient consent statement

All participants have provided written informed consent.

## Ethics approval statement

This study was approved by the Ethics Committee of West China School of Stomatology, Sichuan University.

## Permission to reproduce material from other sources

No material from other sources.

## Clinical trial registration

No clinical trial.

## Funding statement

The authors gratefully acknowledge support from the 10.13039/501100001809National Natural Science Foundation of China (10.13039/501100001809NSFC) (Grant Nos. 82470984, 82271035, U25A6003, 82330029, 32200577), 10.13039/501100012166National Key Research and Development Program of China (Grant Nos. 2022YFC2402901), for financial support. We would like to thank Ninghan Tang (State Key Laboratory of Oral Diseases, West China Hospital of Stomatology, Sichuan; University) for her assistance on confocal laser scanning microscope/slideview. All animal experiments are conducted in accordance with the guidelines outlined in the “Principles of Laboratory Animal Care” and were approved by Medical Ethics Committee of West China Stomatology Hospital, Sichuan University.

## Declaration of competing interests

The authors declare that they have no known competing financial interests or personal relationships that could have appeared to influence the work reported in this paper.

## Data Availability

Data will be made available on request.
