# Supplementary material for: Nucleoside drives the self-assembly and enables delivery of phosphate containing therapeutics
Source: Mater Today Bio. 2026 Aug 13;40:103556. doi: 10.1016/j.mtbio.2026.103556 (PMC13524485; doi:10.1016/j.mtbio.2026.103556)
Supplement: Supplementary file 1 [file mmc1.docx]

**Nucleoside drives the self-assembly and enables delivery of phosphate containing therapeutics**

**Authors:** **Jiamin YAO, Liu LIU, Junfeng WANG, Kaichao WANG, Jiang LIU, Yao YUAN*, Hang ZHAO**

**Affiliations:**

State Key Laboratory of Oral Diseases & National Center for Stomatology & National Clinical Research Center for Oral Diseases & Research Unit of Oral Carcinogenesis and Management & Chinese Academy of Medical Sciences, West China Hospital of Stomatology, Sichuan University, Chengdu 610041, Sichuan, China

* Corresponding author Please contact Yao YUAN: yaoyuan_sklo@scu.edu.cn.

**Fig.S1**

**(a)** Representative images after nucleosides and phosphate-based molecules reaction. **(b)** Chemical structural and hydrogen position of isoG Fig.1d and Fig.1e. **(c)** ^1^H NMR spectra of A, G, C and their 5’-OH phosphate substituents. **(d)** FITR spectra of F-araAMP, isoG and F-araAMP-isoG mixture. **(e)** The electrostatic potential distribution diagrams of F-araAMP and isoG. **(f)** Different perspectives of isoG 5’-OH and F-araAMP phosphate group binding sites.

**Fig.S2**

**(a)** Chemical structure of isoG and PPA. **(b)** The swirling radius of the stable structure. **(c)** ^31^P NMR spectra of F-araAMP and F-araAMP-isoG NSN. **(d)** The molecular structure of isoG and F-araAMP. **(e)** HRMS of F-araAMP-isoG complex. **(f)** HRMS of F-araAMP-*d*isoG complex. **(g)** UV bsorption spectra of F-araAMP-isoG NSN. **(h)** Zeta potential of F-araAMP-isoG NSN. **(i)** CD spectra of F-araAMP-isoG NSN.

**Fig.S3**

(a) The changes solubility of G and A after reacting with phosphate molecules in aqueous solution. **(b)** TEM of F-araAMP-pyrimidine nucleoside NSN. **(c)** TEM of F-araAMP-deoxynucleoside NSN. **(d)** TEM of isoG-siRNA NSN and isoG-oligonucleoside NSN.

**Fig.S4**

(**a**) Schematic illustration of the molecular recognition mechanism of F-araAMP-isoG-NSN. (**b**)The structure and HRMS of Cy5-F-araAMP. (**c**) Representative CLSM image of CAL-27 cells treated with Cy5-loaded F-araAMP-isoG NSN for different time. (**d**) Time-dependent profiles of F-araAMP-isoG NSN fluorescence intensity in the CAL-27 cells by flow cytometry analysis. (**e**) Flow cytometry analysis for apoptosis of CAL-27 induced by F-araAMP, isoG, F-araAMP-isoG mixture, and F-araAMP-isoG NSN at the same drug concentration (300 nM) for 48 h. (**f**) Cell cycle distribution histograms of CAL-27 induced by F-araAMP, isoG, F-araAMP-isoG mixture, and F-araAMP-isoG NSN at the same drug concentration (300 nM) for 48 h. (**g**) Influence of storage on the diameter of F-araAMP-isoG NSN (n=3). (**h**) Representative plasma concentration−time profiles of F-araAMP and F-araAMP-isoG NSN after intravenous administration in rats (n=3).

**Fig.S5**

(a) HE staining of important organs in different treatment groups. **(b)** Body weight monitoring of buccal PDX mice on day 24 (n=5). **(c)** Body weight monitoring of buccal PDX mice on day 20 (n=4). **(d)** Representative HE and IHC staining images of buccal PDX tumor after different treatment. **(e)** IHC analysis of cleaved caspase-3 (n=3).

**Fig.S6**

**(a)** Representative HE and IHC staining images of CAL-7 CDX tumor after different treatment and IHC analysis of Ki67(n=3).
